# Synthetic Polymer Contamination in Bottled Water

**DOI:** 10.3389/fchem.2018.00407

**Published:** 2018-09-11

**Authors:** Sherri A. Mason, Victoria G. Welch, Joseph Neratko

**Affiliations:** Department of Chemistry, State University of New York at Fredonia, Fredonia, NY, United States

**Keywords:** plastic pollution, microplastic, consumables, human health, FTIR, Nile Red, drinking water

## Abstract

Eleven globally sourced brands of bottled water, purchased in 19 locations in nine different countries, were tested for microplastic contamination using Nile Red tagging. Of the 259 total bottles processed, 93% showed some sign of microplastic contamination. After accounting for possible background (lab) contamination, an average of 10.4 microplastic particles >100 um in size per liter of bottled water processed were found. Fragments were the most common morphology (66%) followed by fibers. Half of these particles were confirmed to be polymeric in nature using FTIR spectroscopy with polypropylene being the most common polymer type (54%), which matches a common plastic used for the manufacture of bottle caps. A small fraction of particles (4%) showed the presence of industrial lubricants. While spectroscopic analysis of particles smaller than 100 um was not possible, the adsorption of the Nile Red dye indicates that these particles are most probably plastic. Including these smaller particles (6.5–100 um), an average of 325 microplastic particles per liter of bottled water was found. Microplastic contamination range of 0 to over 10,000 microplastic particles per liter with 95% of particles being between 6.5 and 100 um in size. Data suggests the contamination is at least partially coming from the packaging and/or the bottling process itself. Given the prevalence of the consumption of bottled water across the globe, the results of this study support the need for further studies on the impacts of micro- and nano- plastics on human health.

## Introduction

Plastic is defined as any synthetic or semi-synthetic polymer with thermo-plastic or thermo-set properties, which may be synthesized from hydrocarbon or biomass raw materials (UNEP, 2016). Plastics production has seen an exponential growth since its entrance on the consumer stage, rising from a million tons in 1945 to over 300 million tons in 2014 (PlasticsEurope, 2015). Some of the features of plastic that make it so attractive from a manufacturing standpoint are of concern when it comes to its environmental impact. It is very light-weight allowing it to be easily transported over long distances, and it is durable being resistant to breakage and biodegradation. Its durability is inherently connected to its chemical structure. Being composed largely, if not entirely, of hydrocarbon chains, the lack of double bonds or other functional groups provides an inherent stability to its molecules, and its synthetic nature means that the vast majority of microorganisms haven't evolved to utilize plastic as a food source. Thus, while plastic will break into smaller and smaller particles via photo-oxidative mechanisms, the fundamental molecular structures of the material change very little throughout that process. Plastics become microplastics become nanoplastics, but they are all plastics, just of increasingly smaller size, allowing them to be more easily ingested and perhaps even cross the gastrointestinal tract to be transported throughout a living organism (Brennecke et al., 2015; Sharma and Chatterjee, 2017).

With the rise in plastics manufacture, there has been an associated rise in plastic pollution of the external environment. The first reports date back to the early 1970's (Carpenter and Smith, 1972) and most famously within the world's oceans (e.g., Moore et al., 2001; Eriksen et al., 2014), but more recently plastic pollution has been found within freshwater lakes, inland seas, rivers, wetlands and organisms from plankton to whales (and nearly every species in between; Eriksen et al., 2013; Baldwin et al., 2016; Horton et al., 2017; Lusher et al., 2017).

As its ubiquity in the external environment has been increasing, this has lead more researchers to investigate various consumables for the presence of plastic. The first such study focused on bivalves intended for human consumption (Van Cauwenberghe and Janssen, 2014). More recent studies have focused on fish (such as anchovies), as well as mussels (Rochman et al., 2015; Tanaka and Takada, 2016; Lusher et al., 2017). Two studies have noted the presence of microplastics within beer (Liebezeit and Liebezeit, 2014; Kosuth et al., 2018). Starting with a 2015 study of Chinese Sea Salt brands, several additional studies have established the presence of microplastics within these human consumables as well (Yang et al., 2015; Iñiguez et al., 2017; Karami et al., 2017; Kosuth et al., 2018). The first-ever investigation of plastic pollution within globally sourced tap water (a total 159 samples from seven geographical regions spanning five continents) was published just earlier this year (Kosuth et al., 2018).

As research into the occurrence of plastic pollution has increased, sampling and analysis methods are continually evolving as well. Within the aqueous environment, volume-reduced (using neuston nets) or bulk sampling followed by density separation, filtration/sieving and visual identification have been the most commonly employed methods (Hidalgo-Ruz et al., 2012). Given the time-consuming nature of these methods of sample processing, as well as the potential for misidentification using visual cues alone, one focus area for plastics pollution research (especially at the micro- and nano- scale) is development of methods for high-throughput with increased polymeric confirmation. Several recent studies have supported the use of Nile Red (NR) as an accurate stain for the rapid detection and quantification of microplastics given its selectivity adsorption and fluorescent properties. Maes et al. (2017) specifically tested the preferential adsorption of NR for polymeric materials relative to common organic (algae, seaweeds, wood and feathers) and inorganic (shells) environmental contaminants. Like Maes et al. (2017) and Erni-Cassola et al. (2017) validated the use of this stain with analysis using FTIR to verify the polymeric content of fluorescing particles. Both of these studies concluded from their efforts that NR can be used for the rapid detection of microplastics without the need for additional spectroscopic analysis (thereby reducing the time needed to analyze an environmental sample). These studies suggest that the adsorption of NR alone is sufficient to identify a particle as polymeric in nature. A conclusion further supported by the inclusion of this method within the recent review of analytical methodologies for microplastic monitoring by Renner et al. (2018).

Here we present a study utilizing Nile Red for the detection of microplastic within 11 globally- sourced brands of bottled water. In total 259 bottles of water from 11 brands were processed across 27 different lots (an identification number assigned by a manufacturer to a particular production unit) purchased from 19 locations in nine countries. For 10 brands we tested 2–3 lots each, while for one brand only one lot was tested. Within each lot, we generally tested 10 bottles (bottle volume 500–600 mL each) from the case. However, for one lot, several bottles from the case were seized by customs allowing only nine bottles to be tested, while for two other lots the volume of water per bottle was significantly greater (0.750–2 L) and thus only four (2 L bottles) or six bottles (750 mL bottles) were processed. One of the bottled water lots was packaged in glass (Gerolsteiner, 750 mL, six glass bottles processed); all other samples were packaged in plastic. All bottles had plastic bottle caps.

## Materials and methods

### Sample collection

Sample lots were procured with an eye to geographic diversity (five continents are represented), size of the national packaged drinking water market (China, USA, Brazil, India, Indonesia, Mexico), and high per captia consumption of packaged drinking water (Lebanon, Mexico, Thailand, USA; Table 1). Leading international brands in this study included Aquafina, Dasani, Evian, Nestle Pure Life, and San Pellegrino. Leading national brands included Aqua (Indonesia), Bisleri (India), Epura (Mexico), Gerolsteiner (Germany), Minalba (Brazil), and Wahaha (China).

**Table 1:** Selected market assessment data utilized to determine the countries of origin and brands tested within this study.

			**Brand sales ranking**		**Country sales ranking in world**
**Brand**	**Parent company**	**Country**	**In country**	**In world**	
Aqua	Danone (France)	Indonesia	1	3	4 (by volume)
Aquafina	Pepsico	USA	2	7	2 (by volume)
Bisleri	Bisleri (Indian)	India	1	10	6 (in sales)
Dasani	Coca-Cola	USA	1	4	2 (by volume)
Epura	Proprietary brand of GEPP	Mexico	1	—	1 (per capita)
Evian	Danone	USA United Kingdom France	1 (UK) 2 (France)	3	1 (in sales)
Gerolsteiner	GmbH & Co. KG	GERMANY	1	—	4 (per capita) 8 (in sales)
Minalba	Edson Queiroz Group	Brazil	—	—	5 (in sales)
Nestle Pure Life	Nestle	Lebanon	1	1 (parent company)	—
San Pellegrino	Nestle	Italy	—	1 (parent company)	3 (per capita) 9 (in sales)
Wahaha	Hangzhou Wahaha Group	China	1	1	1 (by volume)

*Dashes (—) indicate missing information*.

As many bottled water brands are simply filtered municipal tap water, sample lots were purchased from a number of locations to increase the likelihood of diverse bottling sources. For example, cases of the Mexican brand Epura were purchased from Tijuana in Baja California state, Reynosa on the Texas border (1,200 miles east of Tijuana), and Mexico City (1,400 miles south of Tijuana). This pattern is repeated with the other brands.

### Sample processing

The bottles within most (9 out of 11 brands) lots came in containers of 500–600 mL per bottle, while two of the brands contained 0.75–2 L per bottle. For those samples with 500–600 mL per bottle, 10 bottles were randomly chosen from the lot, while for the 750 mL samples, six bottles were chosen, and for the 2 L sample, four bottles were randomly chosen, and placed under a laminar flow fume hood. While under the fume hood, each bottle was opened and injected with a specific volume of Nile Red solution (prepared in acetone to 1 mg mL−1) to yield a working concentration of 10 ug mL−1 (Maes et al., 2017) and re-capped. Nile Red adsorbs to the surface of plastics, but not most naturally occurring materials, and fluoresces under specific wavelengths of light (Erni-Cassola et al., 2017). Bottles were allowed to incubate with the injected dye for at least 30 min. The bottled water was then vacuum filtered through a glass fiber filter (Whatman grade 934-AH, 55 mm diameter, 1.5 um pore).

Filters were examined under an optical microscope (Leica EZ4HD, 8–40 × zoom, integrated 3 Mpixel camera) using a blue crime light (Crime-Lite 2, 445–510 nm, Foster & Freeman) to elicit fluorescence, which was visualized through orange filter viewing googles (Foster & Freeman, 529 nm). All particles larger than ~100 um (which are large enough to be visible to the naked eye and manipulated with tweezers) were photographed, enumerated and typed with respect to morphology (Fragment, Fiber, Pellet, Film, or Foam). Additionally the first 3–5 particles were analyzed via FTIR (PerkinElmer Spectrum Two ATR; 450 cm−1 to 4,000 cm−1, 64 scans, 4 cm−1 resolution; ATR correction) to confirm polymeric identity (Spectrum 10 software suite).

After removal of all particles >100 um, the filter with fluorescing particles was photographed (8 × zoom) through an orange camera filter (Foster & Freeman, 62 mm diameter, 529 nm) in four separate quadrants. To ensure no overlap of the quadrant photographs identification marks were made on the filters prior to turning the filter 90 degrees to take the subsequent photo. In fact, given the zoom factor of the microscope, quadrant photos did not obtain full (100%) coverage of the filter. Each photographed quadrant was analyzed using a software program entitled “Galaxy Count” developed by a former astrophysicist for this specific purpose and briefly described here. Given the fluorescing particles relative to the non-fluorescing background, “Galaxy Count” is able to enumerate the number of particles (as bright spots) in order to quantifying the number of smaller microplastics. To do this, the operator of the software sets a threshold value which is used to convert the quadrant images to black (background filter) and white (fluorescing particles). The software then digitally counts the number of white spots (“stars”) against the dark background (“the night sky”). At the 8 × magnification in which the quadrant photos were taken, 1 pixel was equal to 6.5 um. Thus, while the filter pore size was 1.5 um, the smallest size particle visualized through the use of the combination of photography and software was 6.5 um. There could certainly be particles smaller than 6.5 um, but the method employed here would not be able to assess their presence. Due to the programmatic setting of the threshold value, all digital counts were conducted by two different researchers working independently of one another to account for possible variability.

Microplastic counts for particles >100 um (referred to as “NR + FTIR confirmed particles”) are reported for each bottle. These particles are the ones that were further analyzed by FTIR and thus the types of polymers are also reported. Smaller microplastic particles (6.5–100 um; referred to as “NR tagged particles”), counted using the “Galaxy Count” software, are similarly reported for each bottle by summing over the four quadrants (each quadrant being reported as the average of the two researchers).

### Quality assurance and quality control

As the “Galaxy Count” software was created specifically for this project in order to verify its accuracy four solutions were created using DI water containing 0, 20, 50 or 100 polyethylene microspheres (Cospheric, PE micropheres, *D* = 1.25 g mL−1, 75–90 um diameter). These solutions were created by one researcher, but processed “blind” by another researcher in a manner identical to the samples themselves (NR injection, incubation, filtering, quadrant photographing and analysis by the “Galaxy Count” software). Additionally the analysis of all filter quadrants by the “Galaxy Count” software for all samples were conducted “blindly” by two separate researchers. These two counts were compared to one another for accuracy, in addition to being averaged for reported numbers.

In order to prevent/reduce potential contamination throughout the sample processing from external sources, such as airborne fibers, work occurred in a laminar airflow cabinet (Mott manufacturing, Phoenix Controls, serviced annually in September) and the workspace was wiped down every week. All glassware was covered with a watch glass when not in use and washed thoroughly between trials. Filters were inspected under a microscope prior to use, and a cotton lab coat and sterling nitrile powder free exam gloves were worn throughout the experimental procedure.

To account for possible lab contamination that could be coming from atmospheric deposition, the chemicals used, the glassware or other aspects of the testing environment, lab blanks containing deionized water (used to wash all glassware) or acetone (used to prepare the Nile Red solution) were processed in a manner identical to the samples themselves. Particle densities within samples were reduced based upon the average densities across all lab blanks.

## Results

### Overview

A total of 259 individual bottles from across 11 different brands and 27 different lots were analyzed for microplastic particulate, subdivided into two size fractions: so-called “NR + FTIR confirmed particles,” which are >100 um, and “NR tagged particles,” which are 6.5–100 um. As quadrant photos did not provide full (100%) coverage of the filter, it is likely that “NR tagged particles” are underestimated. Since individual bottles contained varied water volumes, from 500 mL to 2 L, absolute counts for each bottle and size fraction were divided by sample volume to calculate (raw) densities of microplastic per liter (microplastic particles/L or MPP/L).

Thirteen lab blanks using laboratory deionized water or acetone were processed using methods identical to those for the bottled water samples. For “NR + FTIR particles” (>100 um) the average density was found to be 4.15 MPP/L, with a range of 0–14 MPP/L, while within the smaller “NR tagged particles” (6.5–100 um) the average density was 23.5 MPP/L, with a range of 7–47 MPP/L. Reported microplastic densities for the bottled water samples are calculated (by size fraction) from raw densities less the average from laboratory blanks (Table 1). If raw densities had less than or equal quantities relative to the laboratory blanks, their values were set to zero. Given that quadrant photos did not obtain full (100%) coverage of the filter and that raw densities were reduced by lab blanks, reported densities are expected to be reasonable but conservative accounting of microplastic contamination. Total densities were calculated by summing across the size fractions (Table 2).

**Table 2:** Microplastic particle densities by bottle and size fraction for each brand and lot number.

			**Microplastics densities (MPP/L) by bottle**																													
			**NR + FTIR confirmed particles (>100 um)**										**NR tagged particles (6.5–100 um)**										**Reported total densities (MPP/L)**									
**Brand**	**Lot number**	**Purchase location**	**1**	**2**	**3**	**4**	**5**	**6**	**7**	**8**	**9**	**10**	**1**	**2**	**3**	**4**	**5**	**6**	**7**	**8**	**9**	**10**	**1**	**2**	**3**	**4**	**5**	**6**	**7**	**8**	**9**	**10**
Aqua	IB 101119	Jakarta, Indonesia	6	8	8	4	11	9	4	9	3	6	127	52	55	57	12	0	0	0	0	0	133	60	62	62	23	9	4	9	3	6
Aqua	BB 311019 08:11 PSRL6	Bali, Indonesia	3	9	1	9	8	8	3	19	26	21	2	37	0	142	0	0	7	602	1,466	4,692	4	47	1	152	8	8	10	621	1,492	4,713
Aqua	BB 311019 09:50 STB1	Medan, Indonesia	0	1	3	9	8	0	6	36	8	0	36	94	30	43	41	0	25	3,687	12	5	36	95	32	52	48	0	31	3,722	20	5
Aquafina	Oct0719 0121PF100375	Amazon.com	10	8	14	8	24	14	20	28	10	14	87	37	74	35	132	313	139	1,268	137	153	96	44	87	42	155	326	158	1,295	146	166
Aquafina	BN7141A04117	Chennai, India	22	22	10	16	4	2	10	10	6	16	127	171	71	94	180	1	253	131	212	389	148	192	80	109	183	2	262	140	217	404
Bisleri	HE.B.No.229 (BM/AS)	Chennai, India	38	28	18	8	8	8	14	10	26	24	76	75	144	37	98	32	50	206	2,163	5,207	113	102	161	44	105	39	63	215	2,188	5,230
Bisleri	MU.B.No.298 (MS/AD)	Mumbai, India	14	8	12	6	8	12	2	12	6	10	66	8	17	125	6	20	0	1,799	0	0	79	15	28	130	13	31	2	1,810	6	10
Bisleri	SO.B.No.087 (AS/LB)	New Delhi, India	0	0	0	0	0	2	4	0	0	0	0	0	0	32	0	0	0	0	0	0	0	0	0	32	0	2	4	0	0	0
Dasani	Oct 0118NHBRB	Amazon.com	22	18	12	4	12	20	8	16	22	14	169	99	292	116	74	130	186	99	168	173	190	116	303	119	85	149	193	114	189	186
Dasani	P18NOV17CG3	Nairobi, Kenya	26	0	8	14	2	0	6	2	2	4	0	7	226	56	8	16	28	1	13	332	26	7	233	69	9	16	33	2	14	335
E-Pura	17.11.18	Mexico City, Mexico	9	11	38	26	31	38	36	4	4	28	2	0	946	1,292	1,167	667	2,232	7	56	268	11	11	983	1,318	1,198	704	2,267	11	60	296
E-Pura	14.10.18	Tijuana, Mexico	18	14	21	9	3	3	0	6	4	1	0	78	12	2	0	12	6	6	6	2	18	92	32	11	3	14	6	12	10	3
E-Pura	09.08.18	Reynosa, Mexico	0	0	0	1	–	–	–	–	–	–	0	0	0	148	–	–	–	–	–	–	0	0	0	149	–	–	–	–	–	–
Evian	PRD 03 21 2017 14:02	Amazon.com	18	18	26	10	38	24	22	20	40	46	239	207	156	176	98	222	105	212	153	148	256	224	181	185	135	245	126	231	192	193
Evian	PRD 05 24 17 11:29	Fredonia, NY, USA	4	0	2	0	0	2	2	2	4	0	253	0	77	29	3	50	1	47	96	15	256	0	78	29	3	51	2	48	99	15
Gerolsteiner	07.142018 2 07.07.2017	Fredonia, NY, USA	10	8	2	10	10	10	20	24	20	36	180	35	45	2	13	56	154	3,431	4,974	5,071	189	42	46	11	22	65	173	3,454	4,993	5,106
Gerolsteiner	NV No. AC-51-07269	Amazon.com	8	11	5	8	12	11	–	–	–	–	3	11	4	173	504	479	–	–	–	–	10	21	9	180	516	490	–	–	–	–
Minalba	FAB: 211017 09:06SP	Sao Paulo, Brazil	4	4	4	0	4	4	2	4	2	0	5	0	14	6	7	17	48	0	79	199	9	4	17	6	11	20	50	4	81	199
Minalba	FAB: 160817 15:05SP	Aparecida de Goiania, Brazil	4	0	8	4	0	6	10	2	15	6	0	0	3	43	0	10	11	5	0	0	4	0	11	47	0	16	20	7	15	6
Minalba	FAB: 091217 16:53SP	Rio de Janeiro, Brazil	2	0	4	0	6	0	0	0	0	39	37	0	2	0	54	0	32	25	479	824	39	0	6	0	60	0	32	25	479	863
Nestle Pure Life	100517 278WF246	Amazon.com	24	38	22	22	28	28	32	28	38	40	101	1,074	30	106	110	1,249	622	1,511	7,322	10,351	124	1,111	51	127	137	1,276	653	1,538	7,359	10,390
Nestle Pure Life	P: 4/11/17 01:34 AZ	Beirut, Lebanon	12	18	18	6	12	12	12	8	6	8	64	136	27	0	27	21	40	57	14	0	75	153	44	6	38	32	51	64	19	8
Nestle Pure Life	730805210A 23:28	Bangkok, Thailand	2	28	8	4	28	8	66	12	18	8	140	147	83	23	398	4	3,461	87	105	60	141	174	90	26	425	11	3,526	98	122	67
San Pellegrino	BBE 11.2018 10	Amazon.com	1	4	4	0	2	2	0	2	2	1	74	29	38	0	27	15	30	6	34	35	74	33	41	0	28	17	30	7	35	36
Wahaha	20171102 1214JN	Jinan, China	9	11	4	26	18	4	1	11	4	3	225	198	65	705	54	26	61	62	34	37	234	209	69	731	71	30	62	73	39	39
Wahaha	20171021 3214GH	Beijing, China	0	0	9	4	4	4	3	21	4	–	178	101	39	9	106	55	42	0	21	–	178	101	48	13	110	60	45	21	25	–
Wahaha	20171103 2106WF	Qingdao, China	4	8	4	1	1	8	4	3	11	1	86	108	44	0	0	158	39	0	87	104	91	116	48	1	1	165	44	3	98	105

*Dashes (—) indicate lots in which < 10 bottles were processed. NR, Nile Red*.

Seventeen bottles out of the 259 bottles analyzed (~7%) showed no microplastic contamination in excess of possible laboratory influence indicating that 93% of the bottled water tested showed some sign of microplastic contamination. The densities of microplastic contamination are quite variable ranging from the 17 bottles with no contamination to one bottle that showed an excess of 10,000 microplastic particles per liter (Table 2). The variabilities seen in the individual bottles, even among the same lot and brand, is similar to what is seen in sampling open bodies of water (Yonkos et al., 2014). Patterns in such sampling can be rather stochastic due to the large number of factors that can affect the occurrence of plastic particles (especially at the microscale), like particle-fluid dynamics, as well as variabilities within the manufacturing process itself, leading to the large variabilities seen within the samples. This erraticism highlights the need for large sample sizes, such as that employed here, in order to average across the variabilities to produce a realistic depiction.

Table 3 provides the mean (by size fraction and total), as well as the minimum and maximum, microplastic densities (in MPP/L) for each lot averaged across all the bottles tested. When averaging across the individual bottles, all 27 lots tested showed some quantity of microplastic contamination (Table 2). Within brands there is significant variability between different lots, which could be owing to a number of factors, such as water source, different bottling facilities, or the conditions and/or length of time involved in shipping from bottling facilities to purchase location. The 17 individual bottles that showed no microplastic contamination in excess of possible laboratory background (Table 1) originated from seven lots (~25%) of the 27 tested. Thus, microplastic contamination was found within all bottles in 75% of the lots analyzed.

**Table 3:** Microplastic densities (MPP/L), by size fractions and total, averaged across all bottles within the same lot.

			**Average microplastic densities (MPP/L)**				
			**NR + FTIR confirmed particles**	**NR tagged particles**	**Total**		
**Brand**	**Lot**	**Purchase location**	**(>100 um)**	**(6.5–100 um)**	**Average**	**Minimum**	**Maximum**
Aqua	IB 101119	Jakarta, Indonesia	6.68	30.4	37.1	3	133
Aqua	BB 311019 08:11 PSRL6	Bali, Indonesia	10.5	695	705	1	4,713
Aqua	BB 311019 09:50 STB1	Medan, Indonesia	6.93	397	404	0	3,722
Aquafina	Oct0719 0121PF100375	Amazon.com	14.8	237	252	42	1,295
Aquafina	BN7141A04117	Chennai, India	11.6	162	174	2	404
Bisleri	HE.B.No.229 (BM/AS)	Chennai, India	18.0	808	826	39	5,230
Bisleri	MU.B.No.298 (MS/AD)	Mumbai, India	8.85	204	213	2	1,810
Bisleri	SO.B.No.087 (AS/LB)	New Delhi, India	0.57	3.15	3.72	0	32
Dasani	Oct 0118NHBRB	Amazon.com	14.6	150	165	85	303
Dasani	P18NOV17CG3	Nairobi, Kenya	6.28	68.3	74.6	2	335
E-Pura	17.11.18	Mexico City, Mexico	22.3	664	686	11	2,267
E-Pura	14.10.18	Tijuana, Mexico	7.76	12.2	20.0	3	92
E-Pura	09.08.18	Reynosa, Mexico	0.21	37.1	37.3	0	149
Evian	PRD 03 21 2017 14:02	Amazon.com	26.0	171	197	126	256
Evian	PRD 05 24 17 11:29	Fredonia, NY, USA	1.51	56.7	58.2	0	256
Gerolsteiner	07.142018 2 07.07.2017	Fredonia, NY, USA	14.8	1,396	1,410	11	5,106
Gerolsteiner	NV No. AC-51-07269	Amazon.com	8.96	195	204	9	516
Minalba	FAB: 211017 09:06SP	Sao Paulo, Brazil	2.56	37.5	40.1	4	199
Minalba	FAB: 160817 15:05SP	Aparecida de Goiania, Brazil	5.30	7.19	12.5	0	47
Minalba	FAB: 091217 16:53SP	Rio de Janeiro, Brazil	5.01	145	150	0	863
Nestle Pure Life	100517 278WF246	Amazon.com	29.8	2,247	2,277	51	10,390
Nestle Pure Life	P: 4/11/17 01:34 AZ	Beirut, Lebanon	11.0	38.2	49.3	6	153
Nestle Pure Life	730805210A 23:28	Bangkok, Thailand	18.0	450	468	11	3,526
San Pellegrino	BBE 11.2018 10	Amazon.com	1.68	28.6	30.3	0	74
Wahaha	20171102 1214JN	Jinan, China	9.10	147	156	30	731
Wahaha	20171021 3214GH	Beijing, China	5.53	61.2	66.7	13	178
Wahaha	20171103 2106WF	Qingdao, China	4.40	62.7	67.1	1	165

*Minimum and maximum densities within the lot are also provided. NR, Nile Red*.

**Figure 1:**
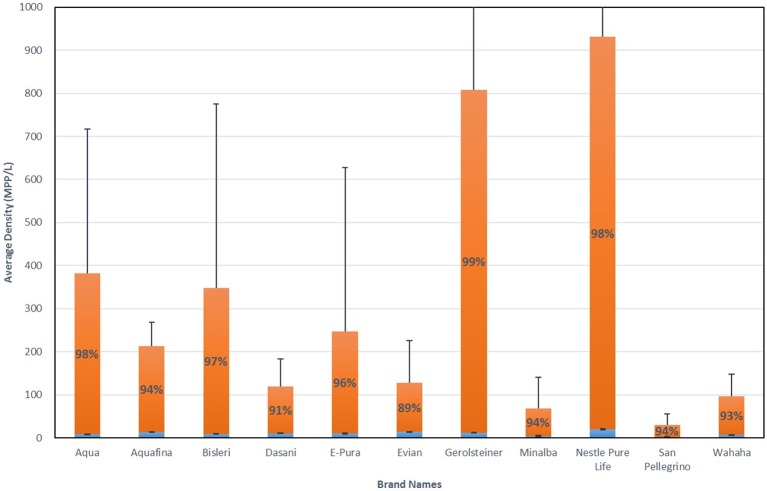
Microplastic density averaged across individual bottles and lots by brand. Blue bars are densities for “NR + FTIR confirmed particles” (>100 um); Orange bars are for “NR tagged particles” (6.5–100 um). Error bars are one standard deviation. Percentages are for the contribution to the total for “NR tagged particles” (6.5–100 um); Contribution of larger particles can be inferred.

When averaged across all lots and all brands, 325 MPP/L were found within the bottled water tested [broken down as an average of 10.4 MPP/L occurring within the larger size range (>100 um) and an average 315 MPP/L within the smaller size range (6.5–100 um)]. While all bottled water lots tested showed some sign of microplastic contamination (Table 2), there was significant variation among the brands (Figure 1). Averaging across lots by brand, Nestle Pure Life and Gerolsteiner showed the highest average densities at 930 and 807 MPP/L, respectively, while San Pellegrino and Minalba showed the lowest microplastic contamination with 30.0 and 63.1 MPP/L, respectively (Figure 1). Error bars in Figure 1 represent one standard deviation and are quite large given the large variability among the individual bottles for each lot (Table 2), as well as the variation among lots of the same brand (Table 3).

Of all the lots tested, only one was packaged in glass rather than plastic: Gerolsteiner (NV No. AC-51-07269). While these samples revealed microplastic contamination, they did so at lower level as compared to the other lots (Tables 2, 3). Further, the same brand of water but packaged in plastic instead of glass was also tested (Gerolsteiner, 07.142018 2). While both of these packaged waters have the same water source, there was considerably less microplastic contamination within the water bottled in glass as compared to that packaged in plastic (204 vs. 1,410 MPP/L, respectively). This indicates that some of the microplastic contamination is likely coming from the water source, but a larger contribution might be originating from the packaging itself.

### NR + FTIR confirmed particles (>100 um)

In total nearly 2,000 microplastic particles >100 um were extracted from all of the filters, with nearly 1,000 (~50%) being further analyzed by FTIR. Obtained FTIR spectra (after applied ATR correction) were compared to libraries of known spectra using the included PerkinElmer Spectrum 10 software suite in order to confirm and identify the polymeric content of the particles. All particles analyzed were either best matched to a polymer, plastic additive or known plastic binder providing additional supporting evidence that Nile Red selectively adsorbed to microplastic particles within the bottled water. With this spectroscopic confirmation, it can be concluded that on average each bottle of water contains at least 10.4 MPP/L (Table 3). While this analysis confirmed the polymeric nature of these particles, a match of 70% or greater was required in order to assign polymer identity. In total over 400 particles (20% of all extracted plastic particles >100 um and 40% of those analyzed by FTIR) met this threshold for identity confirmation and only those results are presented here

**Figure 2:**
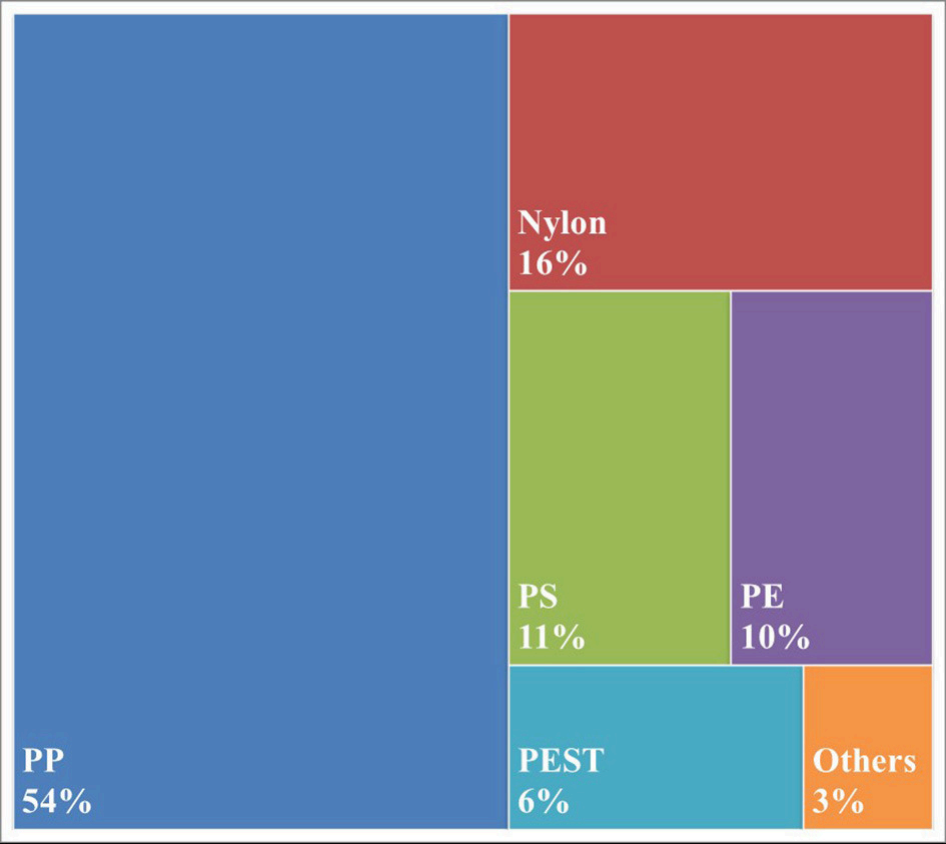
Polymeric content of microplastic particles >100 um found within bottled water. PP, polypropylene; PS, polystyrene; PE, polyethylene; PEST, polyester + polyethylene terephthalate; Others includes Azlon, polyacrylates and copolymers.

Polypropylene was found to be the most common polymeric material (54%) with Nylon being the second most abundant (16%; Figure 2). Polypropylene is a polymer often used to make plastic bottle caps, along with polyethylene, which corresponded to 10% of the particles analyzed. Interestingly, 4% of retrieved particles were found to have signatures of industrial lubricants coating the polymer (not shown).

**Figure 3:**
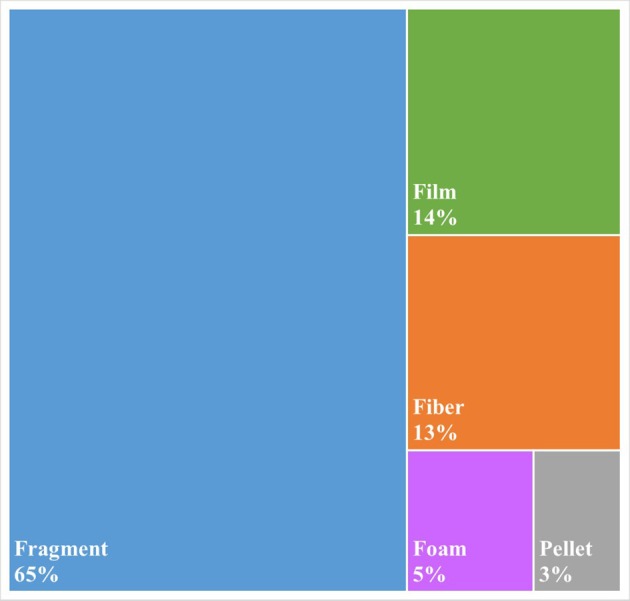
Morphologies of microplastics >100 um found within bottled water.

As is common practice in plastic pollution research, all microplastics >100 um were visually characterized according to their morphology: Fragment, Fiber, Pellet, Film, or Foam. Fragments were found to be the most common type of particle (66%), followed by fibers (13%) and films (12%; Figure 3). The 13% of particles described as fibers (Figure 3) compares well with the 17% of particles that were confirmed by FTIR to be composed of fiberous polymers, most notably Nylon (Figure 2).

### NR tagged particles (6.5–100 um)

**Figure 4:**
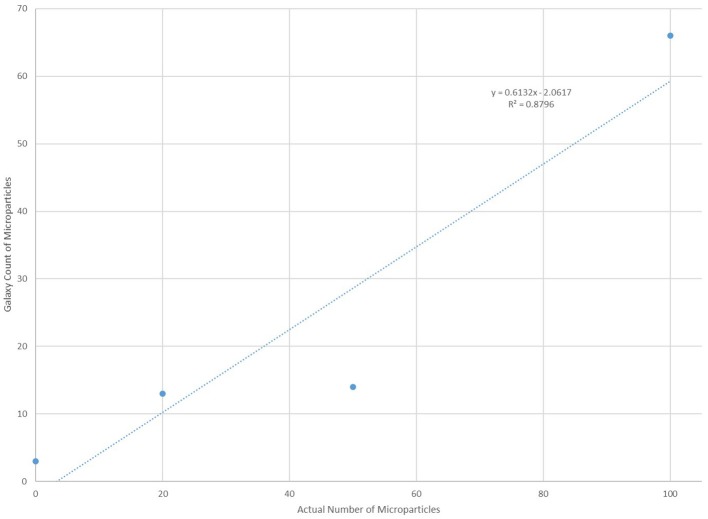
Comparison of counts using the “Galaxy Count” software relative to the known number of microplastic particles within four test solutions.

In order to verify the effectiveness of the “Galaxy Count” software to count microplastics smaller than ~100 um, the software was tested using solutions with known quantities (0,20, 50 or 100) of microspheres (diameters 75–90 um) processed in a manner identical to all samples and lab blanks. The “Galaxy Count” of fluorescing particles on the filter quadrant photos agreed very well with the actual count of particles included within the solutions (Figure 4). The excellent agreement with these test solutions supports the use of this tool for quantifying the numbers of smaller particles within the bottled waters analyzed, while the y-intercept of the least-squares fit further supports that the study is likely undercounting particles, especially within this smallest size range.

**Figure 5:**
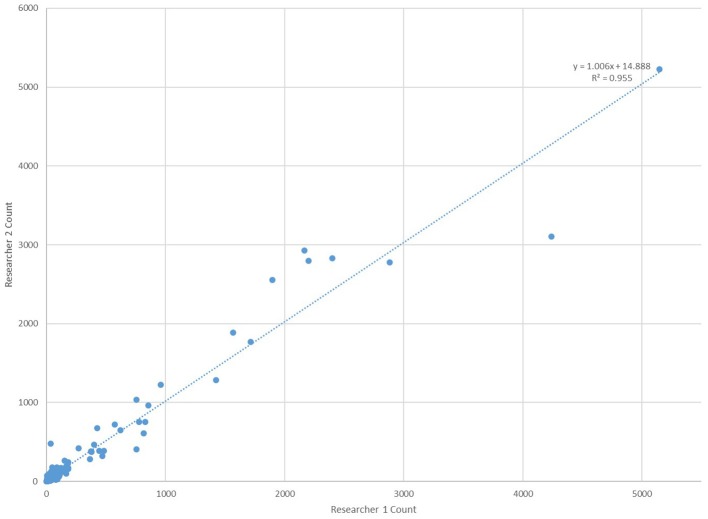
Comparison of microplastic counts by the “Galaxy Count” software for particles <100 um within all 259 bottles tested by two researchers working independently of one another.

All counts using the “Galaxy Count” software were conducted independently by two different researchers owing to possible variabilities in software settings. As shown in Figure 5, the agreement in counts between the two researchers is excellent providing additional support to the effectiveness and validity in using the software to count the smaller particles within the bottled water.

Given the limitations of the lab, particles <100 um (the so-called “NR tagged particles”) were not able to be confirmed as polymeric through spectroscopic analyses (FTIR and/or Raman spectroscopy). However, in testing of various stains and dyes that could be employed for microplastic detection and analysis within environmental samples with a greater potential for misidentification and false positives (i.e., sediments and open-water environmental samples) both Maes et al. (2017) and Erni-Cassola et al. (2017) concluded that Nile Red (NR) was very selective, especially within the time scales of incubation employed, and could be used for the rapid detection of microplastics without the need for additional spectroscopic analysis. To be sure that is why this stain was employed for this study. Additionally FTIR analysis was done on fluorescing particles >100 um and every particle analyzed was confirmed to be polymeric. Even further, NR is well-established to selectively adsorb to hydrophobic (“water-fearing”) materials and, as such, will not adsorb to the only contents reasonably expected to be within bottled water, water and/or its mineral components. In addition, Schymanski et al. (2018) reported Raman confirmed densities of particles within a similar size range and even smaller (5–500 um) in bottles of German bottled mineral water. Thus, at a minimum while particles <100 um were not spectroscopically confirmed to be microplastics, particles are rationally expected to be plastic or of some other anthropogenic origin.

## Discussion

Part of the impetus for this study was as a follow-up to a tap water study released (in part) in September 2017 (Kosuth et al., 2018). The methods used in this study differed slightly in comparison to this earlier study, most notably in the use of a different stain. Rose Bengal was used in the earlier study, while Nile Red was used here. The two dyes have opposite affinities. While plastics adsorb Nile Red (allowing their easy detection via fluorescence), they do not adsorb Rose Bengal. The affinity of plastics to adsorb Nile Red allows smaller particles to be detected as compared to the Rose Bengal method, as noted by a recent study by Erni-Cassola et al. (2017). Thus, only our data on particles >100 um is comparable to the data in this previous tap water study.

We found roughly twice as many plastic particles (>100 um) within bottled water as compared to tap water on average (10.4 vs. 5.45 particles/L). While fibers made of 97% of the microplastics within the tap water study, they only composed 13% of the particles within bottled water. Instead fragments were the most common particle morphology (65%) within bottled water. These results indicate that the main source of the microplastic particulate is different. Given the fragment morphology combined with the fact that 4% of the particles were found to have signatures of industrial lubricants, the data seems to suggest that at least some of the plastic contamination may be coming from the industrial process of bottling the water itself. As polypropylene was the most common polymer found, the fragments could also be breaking off the cap, even entering the water through the simple act of opening the bottle.

More recently Schymanski et al. (2018) published their study on microplastic contamination of packaged mineral water. They tested a wider variety of packaging media from returnable and single-use plastic bottles to cartons to glass, while this study almost exclusively focused on single-use plastic bottles (having only one lot packaged in glass as an alternative). They did test fewer bottles overall as compared to this study. In order to compare these two studies, then, only their data for single-use, plastic beverage bottles is utilized. Within those confines, they tested a total of 11 bottles in comparison to our 259. While they do not specify how many different brands, for one brand they tested two different lots (purchased 6 weeks apart), but only tested one lot for the others.

The average microplastic density across all brands, lot numbers and bottles analyzed (325 MPP/L) is significantly higher in this study as compared to that reported by Schymanski et al. (2018) (14 MPP/L). This difference could be owing to a number of factors. First, as they report they only counted particles for which they could fully confirm the polymeric nature using Raman spectroscopy. We used the adsorption of Nile Red as our frontline confirmation of microplastic identity, using FTIR on particles simply to provide more information as to the specific polymer. As the authors note, while Raman can analyze smaller particles than FTIR, the laser intensity can cause the particle to decompose before an adequate spectra can be obtained. Schymanski et al. (2018) did not include these particles in their counts leading to a reduction in their calculated densities. Further, as our data shows there can be substantial variability between brands and between lots. Our significantly larger sample set provides a greater accounting of that variability.

Another difference between our studies is distribution of polymer types. Schymanski et al. (2018) found PEST (the combination of polyester and polyethylene terephthalate) to be the dominant polymeric material of their particulate contaminants, while that same categorization only accounted for 6% of our analyzed particles. Here polypropylene was found to be the dominant plastic (54%), which only accounted for 1% of their particles. However, our two studies are not fully comparable with regard to this analysis. Schymanski et al. (2018) analyzed and determined polymeric identity for all particles counted, while we only did so for particles >100 um. It is quite possible that the smaller particles we were unable to analyze were mainly composed of the polymers within the PEST category, which would very much alter our percentages. Nevertheless, we both do reason from our data that the packaging of the water itself is a likely source of contamination, though for us it appears to be the caps, while for Schymanski et al. (2018) it appeared to be the bottle.

Despite the differences between our studies some similarities do exist. We both found polyethylene accounting for ~10% of the polymeric contaminants. Additionally, we both found smaller particles provided a larger contribution to the total number of particles as compared to the larger particles (>100 um). Across all samples, 95% of our particles were <100 um, while Schymanski et al. (2018) found they accounted for 98% of their counts. Even further, taken together, these two studies do support the very basic point that there are microplastics within bottled water and at least some of this contamination may arise from the industrial process of bottling the water, as well as from the packing material itself.

## Conclusions

Twenty-seven different lots of bottled water from 11 different brands purchased in 19 locations across nine different countries were analyzed for microplastic contamination using a Nile Red stain, which adsorbs to polymeric material and fluoresces under specific wavelengths of incident light. The use of the fluorescent dye allowed for smaller particles to be detected as compared to a similar study of tap water using a Rose Bengal stain, though the analytical methods employed for their enumeration restricted the lower size limit to 6.5 mm.

Of the 259 total bottles analyzed, 93% showed signs of microplastics. There was significant variation even among bottles of the same brand and lot, which is consistent with environmental sampling and likely resulting from the complexities of microplastic sources, the manufacturing process and particle-fluid dynamics, among others. As bottle volume varied across brands, absolute particle counts were divided by bottle volume in order to produce microplastic particle densities that were comparable across all brands, lots and bottles. These densities were reduced by lab blanks in order to account for any possible contamination. Given our use of lab blanks, the inability to photograph the full filter, the lower limit of one pixel being equivalent to 6.5 mm, and control runs of the software employed to digitally count particles <100 mm, the numbers reported here are very conservative and likely undercounting, especially with regard to smaller microplastics (<100 mm), which were found to be more prominent (on average 95%) as compared to particles >100 mm (on average 5%).

Infrared analysis of particles >100 mm in size confirmed microplastic identity and found polypropylene to be the most common (54%) polymeric material (at least with regard to these larger microplastics), consistent with a common plastic employed to manufacture bottle caps. Smaller particles (6.5–100 mm) could not be analyzed for polymer identification given the analytical limits of the lab. While these smaller particles could not be spectroscopically confirmed as plastic, Nile Red adsorbs to hydrophobic (“water-fearing”) materials, which are not reasonably expected to be naturally found within bottled water. Our FTIR analysis of larger (>100 um particles) fluorescing particles, all of which were confirmed to be polymeric, provides additional support of the selective binding of NR to microplastic particles within the samples. Even further, Schymanski et al. (2018) did spectroscopically confirm (via Raman) particles within this smaller size range in German bottled water as being polymeric in nature provide additional support for their presence. Given this and following the conclusions of prior studies (Erni-Cassola et al., 2017; e.g., Maes et al., 2017) the adsorption of Nile Red alone was used to confer microplastic identity to these smaller particles. As the specific polymer content could not be determined, they could very well show a different compositional pattern as compared to the larger particles analyzed. This could explain the difference in our polymeric compositional analysis relative to a very recent and similar analysis of bottled mineral waters by Schymanski et al. (2018), which found PEST (polyester+polyethylene terephthalate) to be the most common polymeric material, consistent with a common plastic employed to manufacture the bottle itself. Either way both studies indicate that at least part of the microplastic contamination is arising from the packaging material and/or the bottling process itself.

Beyond the polymeric identity of the microplastics, the morphology of the particles also provides an indication as to a different source of contamination relative to an earlier study on globally sourced tap water. In this prior study 83% of the 159 samples were show to contain anthropogenic debris and 98% of those particles were microfibers. In comparison, this study found microplastic contamination within 93% of the individual bottles (and in all of the brands and lots tested) with only 13% of the particles being categorized as microfibers. The vast majority (65%) of the microplastics were identified as fragments indicating a different source of the contamination relative to the tap water. Even further, the bottled water contained on average nearly twice as much microplastic contamination (within the same size range, i.e., >100 um) as compared to tap water (10.4 vs. 5.45 particles/L). While the impacts of microplastic contamination on human health are still unknown, these results strongly support a reduction in the bottling of water and in the consumption of bottled water, especially within locations in which clean, safe tap water exists.

## Data availability

The raw data supporting the conclusions of this manuscript will be made available by the authors, without undue reservation, to any qualified researcher.

## Author contributions

SM designed the study, supervised the work, ensured quality control and wrote the manuscript. VW was the lead laboratory research assistant and conducted all aspects of the laboratory analysis. JN assisted in and conducted laboratory analyses.

### Conflict of interest statement

The authors declare that the research was conducted in the absence of any commercial or financial relationships that could be construed as a potential conflict of interest.
